# Influencing factors of presenteeism among Portuguese workers in a private social solidarity institution in the aftermath of COVID-19

**DOI:** 10.1192/j.eurpsy.2024.1042

**Published:** 2024-08-27

**Authors:** C. Laranjeira, A. C. Maurício

**Affiliations:** ^1^ School of Health Sciences; ^2^ciTechCare, Polytechnic University of Leiria, Leiria; ^3^Santa Casa da Misericórdia de Porto de Mós, Porto de Mós, Portugal

## Abstract

**Introduction:**

In ordinary circumstances, the employed individuals demonstrate significant levels of psychological distress and presenteeism, a situation that has been further intensified by the COVID-19 epidemic. Furthermore, a limited number of studies have examined the phenomenon of presenteeism in the context of the COVID-19 pandemic, therefore necessitating the undertaking of the present study.

**Objectives:**

The goal of this study was to investigate the levels of presenteeism and its related characteristics, as well as job satisfaction and psychological distress, among a sample of employees employed at a Private Social Solidarity Institution (IPSS) in Portugal.

**Methods:**

A cross-sectional survey was undertaken in 2022 to observe personnel from an IPSS located in the central area of Portugal. The research had a sample size of 71 workers who were provided with a signed authorization. The survey was designed to gather both general and professional information from participants. Additionally, it included the Stanford Presenteeism Scale (SPS-6), the Job Satisfaction Questionnaire (S20/23), and the Kessler Psychological Distress Scale (K10) as measurement tools.

**Results:**

The occurrence of presenteeism was seen in 32 employees, accounting for 45.1% of the sample, whereas illness absence was reported by 38 workers, representing 54.3% of the sample. The majority of the individual assessments for S20/23 demonstrated a higher degree of satisfaction (mean ≥ 4.5 points.), with the exception of the salary-related issue, which elicited a higher level of discontent (mean = 3.36 ± 1.9 pts.). Approximately 50.7% of the individuals had a high or very high susceptibility to experiencing or developing a mental condition. The correlation matrix revealed a statistically significant moderate positive association between presenteeism and work satisfaction, as well as a statistically significant moderate negative link between presenteeism and psychological distress (p<0.01). The associated factors of presenteeism were found to be marital status, quality of sleep, illness absenteeism, health perception, and psychological distress. The combined effect of these predictors was shown to account for 35.8% of the variance in presenteeism.

**Conclusions:**

It is expected that the outcomes of our research will stimulate more investigations into the tangible implications of presenteeism in promoting improved health and well-being within the workplace.

**Disclosure of Interest:**

None Declared

